# Temperature and microwave near field imaging by thermo-elastic optical indicator microscopy

**DOI:** 10.1038/srep39696

**Published:** 2016-12-22

**Authors:** Hanju Lee, Shant Arakelyan, Barry Friedman, Kiejin Lee

**Affiliations:** 1 Department of Physics and Basic Science Institute for Cell Damage Control, Sogang University, Seoul 121-742, Republic of Korea; 2 Department of Radiophysics, Yerevan State University, Yerevan 0025, Armenia; 3 Department of Physics, Sam Houston State University, Huntsville, TX 77341, USA

## Abstract

A high resolution imaging of the temperature and microwave near field can be a powerful tool for the non-destructive testing of materials and devices. However, it is presently a very challenging issue due to the lack of a practical measurement pathway. In this work, we propose and demonstrate experimentally a practical method resolving the issue by using a conventional CCD-based optical indicator microscope system. The present method utilizes the heat caused by an interaction between the material and an electromagnetic wave, and visualizes the heat source distribution from the measured photoelastic images. By using a slide glass coated by a metal thin film as the indicator, we obtain optically resolved temperature, electric, and magnetic microwave near field images selectively with a comparable sensitivity, response time, and bandwidth of existing methods. The present method provides a practical way to characterize the thermal and electromagnetic properties of materials and devices under various environments.

Miniaturization, integration and increased switching speeds of electronic devices have led to tremendous progress in information and communication technologies. Modern electronic devices are miniaturized from a few micrometers to a few tens of nanometer scales, and their operation speeds reach up to the microwave frequency region. At the same time, problems such as localized heating, electromagnetic interference and compatibility (EMI&C) in these devices have become critical issues that limit their performance and reliability^1^. Achieving a high resolution spatial distribution of the temperature and microwave near field (MWNF) in such miniaturized devices will provide a practical way to diagnose, characterize and resolve the thermal and EMI&C problems, however, it is currently very challenging due to the lack of a practical measurement pathway^4^,^5^.

Various approaches have been proposed based on different kinds of physical phenomena^4^,^5^,^6^,^7^,^8^,^9^,^10^,^11^,^12^,^13^. They often rely on scanning probe techniques with carefully designed scanning probes, particular materials and physical processes. These features limit their practicality as they require commercially unavailable and expensive materials and instruments with extreme measurement conditions such as a long time stability, temperature, strong external magnetic field, and so on. In particular, while the scanning techniques provide outstanding spatial resolution reaching up to a few tens of nanometer scales^3^, their long measurement time is a common drawback that limits their practical use. Scanning free approaches utilizing optical imaging array sensors, such as a charge coupled device (CCD) or complementary metal–oxide–semiconductor (CMOS) imaging sensor, may resolve the issue of the long measurement time of the scanning techniques^14^,^15^,^16^. However, the proposed approaches have common limitations in that commercially unavailable and expensive materials and instruments, and particular measurement conditions are required.

Here, we report a new optical indicator method, thermo-elastic optical indicator microscopy (TEOIM), for the high resolution imaging of the thermal and microwave near field distribution. The measurement principle of TEOIM is based on a reconstruction of the heat source distribution of the optical indicator (OI) from its thermal stress distribution obtained by photoelastic measurement. Depending on the experimental configuration and material property of the OI, the visualized heat source distribution can describe a transferred heat from the DUT by a direct heat conduction process, a generated heat by an absorption of the infrared (IR) light radiated from the DUT, and a generated heat by an interaction between the OI-material and the electric (E-) or the magnetic (H-) MWNF. The advantages of TEOIM are that it requires conventional device and material, such as a CCD-based polarized light microscope and a typical slide glass, and the measurement principle is based on a generally occurring physical phenomenon in that heat is always involved whenever a material interacts with an electromagnetic wave. The simplicity of the measurement system and the generality of the measurement principle are attractive features for the implementation of a practical measurement system resolving problems of previous methods. In this paper, we discuss the measurement performance of TEOIM such as spatial resolution, response time, bandwidth, and sensitivity. We present examples demonstrating that TEOIM provides a comparable measurement performance to that of existing methods, a capability of visualizing the temperature and the MWNF distribution for various experimental conditions.

## Results

### Measurement principle

**Figure 1:**
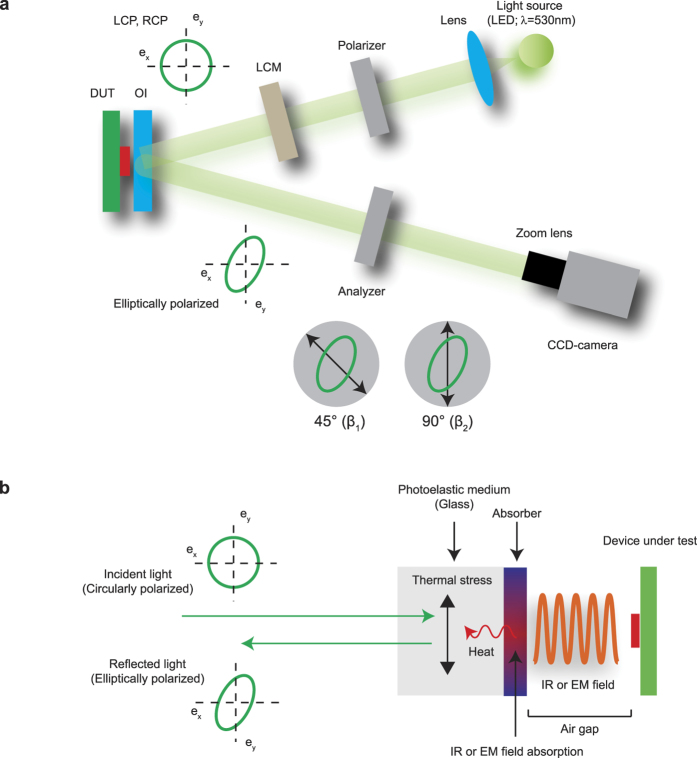
Measurement principle of TEOIM. (**a**) Illustration of measurement principle of the TEOIM system. A circularly polarized incident light changes to an elliptically polarized state by the photoelastic effect of the optical indicator (OI) caused by a thermal stress. The thermal stress comes from the electromagnetic heating caused by interaction between the OI-material and electromagnetic wave radiated from a device under test. (**b**) Illustration of experimental setup of TEOIM. The incident light is modulated to be circularly polarized (left handed polarized: LCP; right handed polarized: RCP) states by the liquid crystal modulator (LCM) and a linear sheet polarizer. The polarization state of the reflected light from the OI is determined by measuring the light intensity passing through the analyzer aligned at 0°and 45°, where the light intensity is measured by charge coupled device (CCD) image sensor.

Figure 1a illustrates the measurement system of TEOIM. The optical indicator (OI) composed of a glass substrate coated by a thin film absorbing the heat, IR or EM field radiated from a device under test (DUT), is placed on the DUT and monitored by a polarized light microscope system (Fig. 1a). The incident light is polarized linearly by a sheet polarizer, and after that, the polarization state is modulated into circularly polarization states (left handed polarized: LCP; right handed polarized: RCP) by a liquid crystal modulator (LCM). The change of the polarization state of the reflected light, which has passed through the OI, is analyzed from a change of the optical intensity transmitting through a sheet polarizer measured using a CCD camera. More detailed information on the polarization modulation and analyzing methods are presented in the method section and the supplementary text S1–S3.

Figure 1b illustrates the measurement principle of TEOIM. When the DUT is in an operating state, it radiates heat, IR or EM fields, and they are absorbed selectively depending on the absorption property of the OI. The generated heat by the absorption diffuses into the thermo-elastic medium (glass substrate) resulting in a thermal stress inside the medium. When a circularly polarized light passes through the stressed medium, the polarization state of the light is changed into an elliptically polarization state depending on the stress axis and material property of the medium. This is known as the photoelastic effect (alternately the stress optical effect), and one can measure the stress distribution by monitoring a change of the linear birefringence (LB) of the medium. Then, one can measure the LB change of the OI caused by the DUT from optical intensity change passing through a linear polarizer aligned 0° and 45° from the vertical direction to the incident plane, as shown in Fig. 1a and supplement text S2^17^,^18^.

The temperature and stress distribution of the OI can be calculated by solving the inverse problem of determining the plane stress and heat conduction from LB measurement results^19^,^20^,^21^,^22^. In general, it requires complicated numerical techniques and methods, and there is no analytical solution that can be applied to a general condition. However, since the heat source distribution is the object to be visualized, the problem is drastically simplified as shown in supplement text S3:


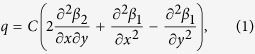


where, *q* is the heat source density, *β*_1_ and *β*_2_ are LB distribution images related to the normal and shear stresses of the OI, and *C* is a constant parameter related to physical properties of the OI and the wavelength of the probing light. The heat source density, *q*, is the heat in the thin film generated by the radiated heat, IR or MWNF, and therefore, the calculated heat source distribution describes the temperature or the MWNF of the DUT.

### Temperature distribution imaging

**Figure 2:**
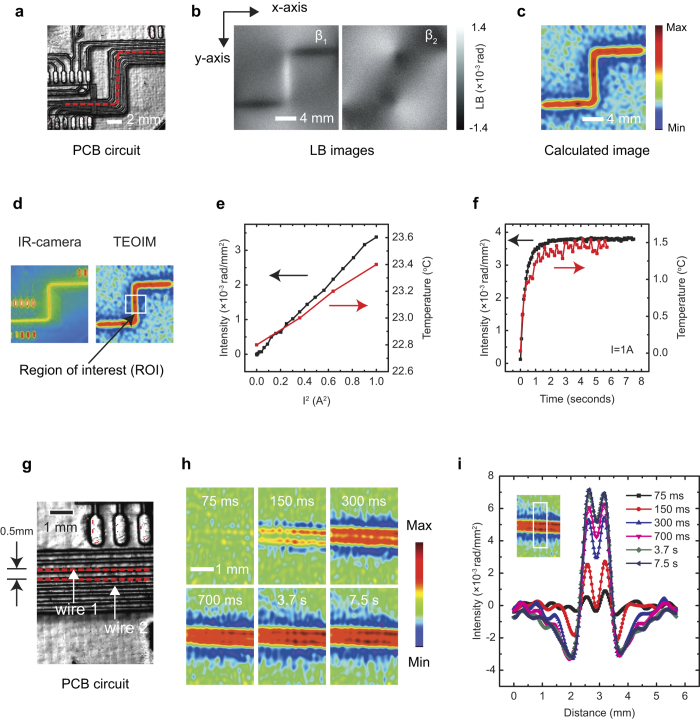
Thermal imaging by TEOIM for a PCB circuit. (**a**) Optical image of the PCB circuit used in the experiment. (**b**) Linear birefringence images of the OI placed on the PCB circuit. (**c**) Calculated heat source distribution image. (**d**) Temperature image measured by infrared (IR) camera and heat source distribution image measured by TEOIM, where the white rectangle indicates the region of interest (ROI) for the temperature sensitivity calculation. **(e,f**) Intensity and temperature changes of PCB circuit around ROI as a function of the applied current (**e**) and time (**f**). (**g**) Optical image of the PCB circuit used for the time resolved imaging, where DC current of 1A was applied to the two wires, and the distance between the wires was 0.5 mm. (**h**) Time resolved heat source distribution images. **(i**) Intensity profiles of heat source distribution images for various time. Inset shows the ROI for the intensity profiles.

To verify the proposed approach, we performed experiments visualizing the temperature distribution of a PCB circuit (Fig. 2a), where a borosilicate glass substrate (Eagle XG; Corning) coated by platinum (pt) thin film (thickness = 200 nm) was used as the OI. The OI was placed on the PCB circuit whose surface was coated by an electrically insulating polymer, where the pt layer of the OI was contacted to the electrically insulated surface of the PCB circuit so that the heat generated by the current in the PCB circuit was transferred to the glass substrate directly. Figure 2b show the LB measurement results related to the normal (*β*_1_) and shear (*β*_2_) components of the thermal stresses. The LB images showed that the normal components of the thermal stress appeared dominant around the straight parts of the conduction line, while shear components of the thermal stress appeared dominant around the curved parts of the conduction line. In the straight parts of the conduction line, the temperature distribution along the line is uniform so that the normal stress perpendicular to the direction of the conduction line is dominant. On the other hand, because two normal stresses parallel to the x-axis and y-axis meet around the curved parts of the conduction line, the shear stress appears dominant around this region. Figure 2c shows the calculated heat distribution image from β1 and β2 images by using the equation (1). As expected, the calculated heat source distribution showed intense regions along the conduction line of the circuit, and it was in good agreement with the temperature image measured by an IR-camera as shown in Fig 2d. Figure 2e,f show intensity changes of the calculated image and temperature in the region of interest (ROI; shown in Fig. 2d) according to the incident power and time, respectively. The calculated intensity increased linearly as a function of applied power to the PCB circuit, and it was in good agreement with the change of temperature of the PCB circuit. The time dependence of the calculated intensity also showed a consistent result with the temperature change as shown in Fig. 2f, where the calculated intensity increased rapidly and saturated consistently with the temperature change of the PCB circuit. Figure 2h,i show calculated heat images and intensity profiles of time resolved measurements results, where 1 A electric current was applied to two conduction wires with spacing 0.5 mm as shown in Fig. 2g. The time resolved images showed that the intensity increased rapidly up to 300 ms after applying the electric current, and it saturated around 700 ms showing a negligible change after this time. The calculated images and their intensity profiles showed spatially well resolved heat distribution of the two conduction wires with a rate of 13 frames per seconds. We note that the response time of the TEOIM will depend on how fast the thermal energy of the DUT is transferred to the OI, and how fast the thermal stress of the OI is saturated for a given temperature change. Therefore, the thermal diffusion process of the OI will limit the response time if the measurement speed is faster than that of thermal diffusion process. As shown in Fig. 2f, the time dependent measurement result by the TEOIM coincided with the IR measurement result, and thus, we assumed that the response time is faster than the frame rate (~80 ms per frames) of our CCD camera.

To identify the temperature sensitivity of the TEOIM, we estimated the noise-equivalent temperature difference (NETD)^5^,^23^. The NETD is the classical measure of the temperature sensitivity of infrared imagers, and is defined as


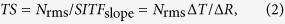


where, *N*_rms_ is the root mean square noise of the background signal of the system, SITF is the system intensity transfer function, *SITF*_slope_ is Δ*R*/Δ*T*, Δ*T* is the target-to-background differential temperature, and Δ*R* is the measured device response. In the present system, the *N*_rms_ corresponds to intensity fluctuations of the calculated image, and Δ*R* is an intensity change of the calculated image for Δ*T*. The *SITF*_slope_ and *TS* were calculated from Fig. 2e, and the *N*_rms_ was calculated by estimating the standard deviation of the background measurement result (*I* = 0 A). For 1,000 times averaged measurements, the estimated value of *SITF*_slope_ and *N*_rms_ were 2.5~3.2 × 10^−3^ rad∙K^−1^ and 3.2~4.4 × 10^−5^ rad respectively, and the *TS* was calculated to be 60~136 mK. It is important to note that the *N*_rms_ depends on the averaging and smoothing procedures of measurements and calculations as shown in supplement text S4. For 10,000-time averaged measurement result, the *N*_rms_ varied from ~10^−6^ to ~10^−7^ depending on the size of the smoothing process, and corresponding *TS* was 4~18 mK.

**Figure 3:**
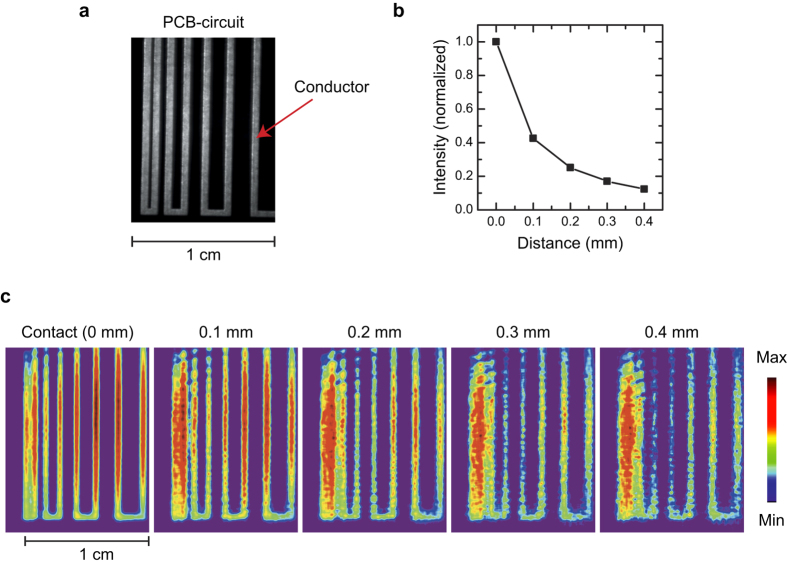
Non-contact thermal imaging for a PCB circuit. (**a**) Optical image of a PCB circuit. (**b**) Calculated intensity changes as a function of distance between the OI and PCB circuit. The calculated intensity was normalized to the intensity measured in the contact configuration. (**c**) Calculated thermal images as a function of distance between the OI and PCB circuit. Each thermal image was normalized to its maximum intensity.

The TEOIM can operate in a non-contact configuration that the OI is separated from the DUT by an air gap. In the non-contact configuration, the heat generated by the DUT is transferred to the OI through the IR rather than the direct heat conduction, where the IR radiated from the DUT is absorbed in the OI resulting in a thermal stress inside the OI. Figure 3 shows a practical example on the non-contact thermal imaging, where the thermal distribution of a PCB circuit (Fig. 3a) under DC current was visualized by the TEOIM for various distances between the PCB circuit and the OI (Fig. 3c). The measured images well describe the thermal distribution of the PCB circuit induced by the Joule heating, and demonstrate the functionality of the TEOIM for the non-contact thermal imaging. In addition, the calculated intensity of the heat source as a function of distance is presented in Fig. 3b, where one can see that the intensity decays exponentially as the distance is increased. When the distance was around 0.5 mm, the calculated intensity decreased to 0.1 compared to the intensity measured in the contact configuration. Therefore, the *TS* in the non-contact configuration with an air gap of 0.5 mm is around 0.6~1.4 K and 5~14 mK for 1,000 and 10,000 times averaged measurements, respectively. The estimated *TS* of the TEOIM is comparable to that of modern thermal image technologies (35–200 mK)^3^,^5^,^6^,^24^. In addition, we present detailed discussion on the spatial resolution of the TEOIM in the supplement text S9. From the discussion, one can see that the spatial resolution of the TEOIM is comparable to that of a typical optical microscope system. For instance, the resolution limit of a traditional optical microscope using a conventional lens system with green wavelength (550 nm) probing light is around 200 nm. Based on the discussion in the supplement text S9, where the relative resolution of the TEOIM to the traditional optical microscope varies from 1.25 to 0.75 depending on an interpolation process, one can expect that the principle resolution limit of the TEOIM using green wavelength light (550 nm) will be around 150~250 nm. Therefore, we can conclude that TEOIM provides comparable *TS*, response time, and spatial resolution to that of modern thermography technologies.

### Microwave near field distribution imaging

As we discussed in the measurement principle section, the physical quantity visualized by TEOIM is determined by the absorption property of the thin film coated on the glass substrate. Therefore, one can visualize a physical quantity by choosing a proper material absorptive to the physical quantity. For the microwave frequency region, there are two different heating mechanisms for a non-magnetic material: dielectric and resistive losses^25^. The dielectric loss is related to the electrical energy dissipation of an insulating material by the oscillating electric field. On the other hand, resistive loss is related to Joule heating of a highly conductive material by an electrical current induced by the oscillating magnetic field. Therefore, the dielectric and resistive losses can be a proper physical phenomenon for the imaging of the electric and magnetic components of the microwave near field, respectively, and a selective imaging can be achieved by coating a material having a high dielectric-loss or a high conductive property on the glass substrate (see the supplement text S5).

**Figure 4:**
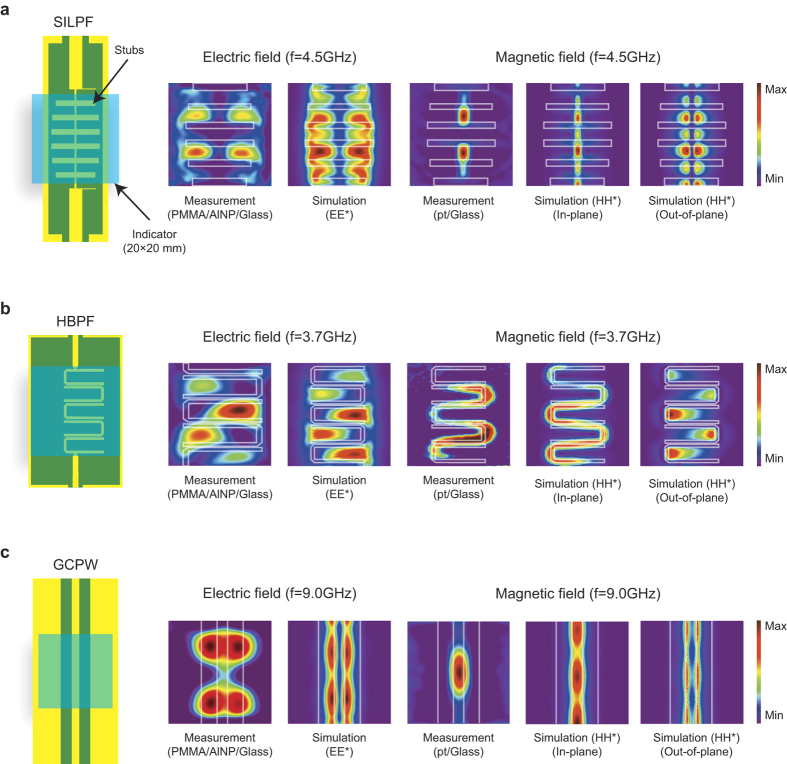
Electric and magnetic microwave near field distribution of SILPF, HBPF, and GCPW . Measured and simulated electric and magnetic components of microwave near field (E-MWNF and H-MWNF) distributions: (**a)** SILPF at 4.5 GHz; (**b**) HBPF at 3.7 GHz; (**c**) GCPW at 9 GHz. The images were normalized for their maximum intensity.

To demonstrate the MWNF distribution imaging by TEOIM, we fabricated indicators composed of a borosilicate glass substrate coated by microwave lossy materials. For the electric field imaging indicator, we used aluminum nanoparticles (AlNP) coated by a poly(methyl methacrylate) (PMMA) thin film because it is known that metal nanoparticles embedded in glass and polymer give a large increase of the dielectric loss constant^26^. On the other hand, a conductive platinum (pt) thin film of 200 nm thickness was used for magnetic field imaging indicator because metal thin films have a large microwave absorptive property and are well heated by the microwave magnetic field^27^,^28^. Figure 4 shows representative measurement and simulation results of MWNF distribution for (a) stepped impedance low pass filter (SILPF), (b) hairpin band pass filter (HBPF), and (c) grounded coplanar waveguide (GCPW), which are commonly used as the building blocks of various microwave devices and systems. From the measurement results, one can see that the measured heat distributions by AlNP-indicator coincide with the E-MWNF distribution of the DUTs. We also tested a bare glass, AlNP, and PMMA coated glass substrates to verify the MW heating process of the indicator. As shown in supplement text S7, while the LB of glass and PMMA/glass samples were nearly unchanged, there was a weak change of LB for the AlNP/glass sample, and it was drastically enhanced by the additional PMMA coating. These results clearly show that the heat is mainly originated from the microwave dielectric heating of AlNPs, and contributions of heat transferred from the DUT by a heat diffusion or IR radiation can be ignored. We note that the subject to be visualized by the TEOIM depends on the dominant process that generates heat resulting in a thermal stress in the OI. For instance, if the temperature of the DUT changed significantly during the measurement so that the thermal stress mainly comes from the IR absorption rather than the MW heating process, the temperature distribution of the DUT will be visualized by the TEOIM. Therefore, the measurement results should be analyzed carefully when the temperature of the DUT is significantly changed, and this can be done by measuring the temperature of the DUT or a reference measurement by using an OI that is inactive to the MWNF such as a bare glass substrate before the MWNF imaging measurement.

Contrary to the AlNP-indicator, the heat distribution images of pt-indicator were coincident with that of the in-plane H-MWNF distribution. This result shows that the microwave heating mechanism of metal nanoparticles and thin films are different, and the heating of metal thin film originates from the Joule heating by the microwave magnetic field. It is important to note that because the electrical current is induced by the microwave magnetic field perpendicular to the surface normal of the metal thin film^27^,^29^, the relative direction between the microwave magnetic field and surface normal of the metal thin film should be considered for the measurement. In addition, we present frequency characteristics of the MWNF structure of the SILPF and HBPF in the supplement text S8. The measurement results showed that a strong enhancement of the MWNF intensity appeared within the pass band frequency, while a strong attenuation appeared when the microwave frequency was out of the pass band. These results well describe the frequency response of the filter devices, and thus, one can investigate the frequency characteristics of microwave devices from visualized MWNF structures.

**Figure 5:**
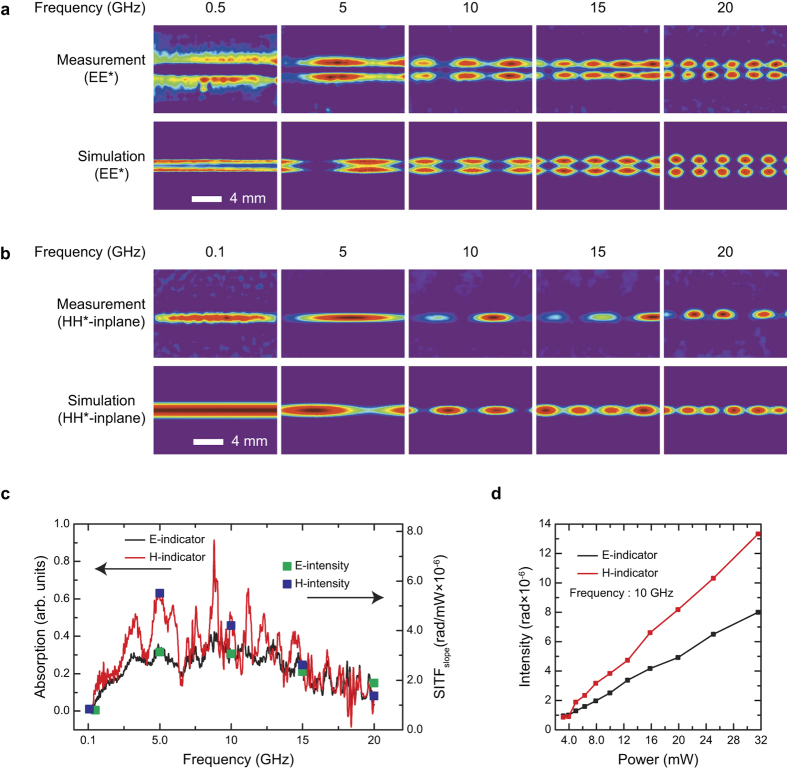
Electric and magnetic microwave near field distribution of microstrip line as a function of microwave frequency. Measured and simulated MWNF distribution of the microsctrip line as a function microwave frequency: (**a**) E-MWNF; (**b**) H-MWNF. (**c**) Microwave absorption spectra of the AlNP- (black line) and pt- (red line) indicators measured by network analyzer. The *SITF*_slope_ of the indicators at 0.1 (0.5 for electric field), 5, 10, 15 and 20 GHz are for electric (green rectangles) and magnetic (blue rectangles) fields are also presented. (**d**) Calculated average intensities of electric (black) and magnetic (red) fields at 10 GHz as a function of incident microwave power.

**Figure 6:**
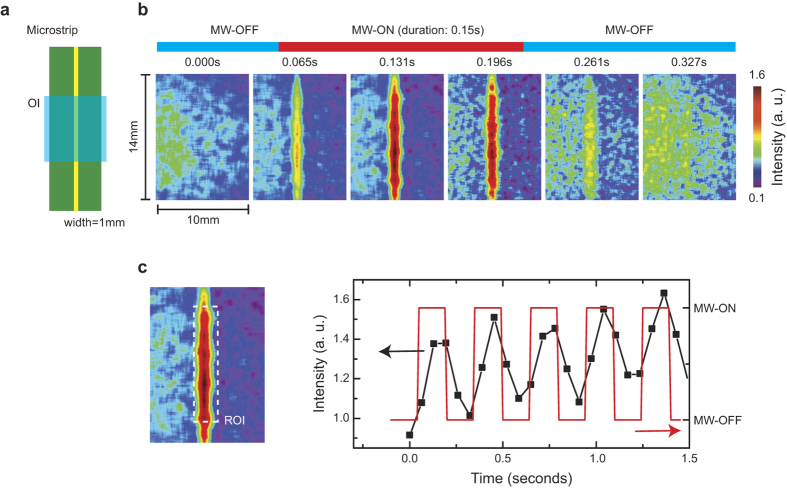
Real time imaging of magnetic microwave near field structures of a micro-strip line at 0.2 GHz. (**a**) Illustration of micro-strip line used in the experiment. (**b**) Real time images of magnetic microwave near field distribution, where a pulse of microwave signal (duration 150 ms) was applied continuously. (**c**) Real time intensity changes of magnetic microwave near field (black) around the region of interest (ROI) as a function of time, where the red line indicates pulses of input microwave signal.

To verify the measurable microwave bandwidth, we conducted MWNF imaging for the microstrip line from 0.1 GHz to 20 GHz. Figure 5 shows measured and simulated E-MWNF (a) and H-MWNF (b) distributions, and an illustration for the microstrip line is presented in Fig. 6a. The lowest measurable microwave frequency of the E-MWNF measurement was around 0.5 GHz, while that of H-MWNF measurement was lower than 0.1 GHz. The measurements results for E- and H-MWNF showed well defined MWNF structures of the microstrip line up to 20 GHz, where the frequency was the maximum frequency provided by our instrument. The simulated microwave electric and magnetic field structure of the microstrip line coincide well with that of measurement results, and thus, we can conclude that the present OIs can visualize the electric and magnetic component of microwave field selectively up to 20 GHz. Because of limitation of our signal generator, we confirmed the bandwidth limit of TEOIM up to 20 GHz. However, we note that because the measurement principle is based on the loss mechanism of a material for the electric and the magnetic field of the electromagnetic wave, the measurable bandwidth can be extended to further higher frequency up to terahertz provided a proper absorptive material is applied to the OI.

As the measurement principle is based on the electromagnetic wave absorption of a material, one can estimate the measurement sensitivity of the TEOIM from the microwave absorption spectrum of the OI. Figure 5c shows the relative absorption spectrum for both of indicators, where one can see that measured intensity values were in good agreement with absorption curve for both indicators. Figure 5d shows calculated intensity changes as a function of incident microwave power at 10 GHz, where one can see that the intensity increases linearly along with an increase of incident microwave power. We estimated sensitivities of the indicators for incident microwave power by using the equation (2), where the *ΔT* is replaced by the microwave power (*ΔP*). The estimated sensitivities at 10 GHz were around 0.1~1 mW for 15,000-times averaged measurements, and this sensitivity was comparable to that of conventional methods. For the electric field imaging, the estimated sensitivity is comparable to that of electro optical effect based scanning free OIM^15^. Otherwise, to the best of our knowledge, there has been no report on the scanning free method for the H-MWNF imaging. The reported sensitivity of the scanning method based on the magneto optical effect with an optical scanning probe is around 0.03 mW^10^. However, this sensitivity is achieved by a noise reduction of the measurement system, and the MO effect by the H-WMNF is extremely weak so that it cannot be detected by a typical CCD camera^16^.

Because the TEOIM is a scanning free approach using a CCD camera, it has a unique advantage in the real-time measurement over scanning based methods (a comparison table for non-scanning probe methods is presented in the supplementary table S2). Figure 6 shows full real-time measurement results on the H-MWNF of a micro-strip line at 0.2 GHz with a power of 5 W, where the measurement were conducted with maximum frame rate of the CCD camera without accumulating and averaging of measured frames. We note that for a high power microwave measurement, the existing methods based on electro-optical (EO) and magneto-optical (MO) effects will have problems caused by an increase of temperature of the OI. The problems arise because the EO and MO effects change as a function of temperature, and the materials can be heated by the strong microwave resulting in changes of MO and EO effects. In particular, because the MO and EO measurements are based on the polarized light microscopy, the photoelastic effects caused by the thermal stress will cause artifacts in the MO and EO signal. However, since the TEOIM utilizes the heating of materials directly, it is free from the heating problems, and it even provides a more intense signal as the applied microwave power increases. Therefore, the TEOIM has a unique advantage in the real-time measurement with a strong microwave power over the existing methods.

**Figure 7:**
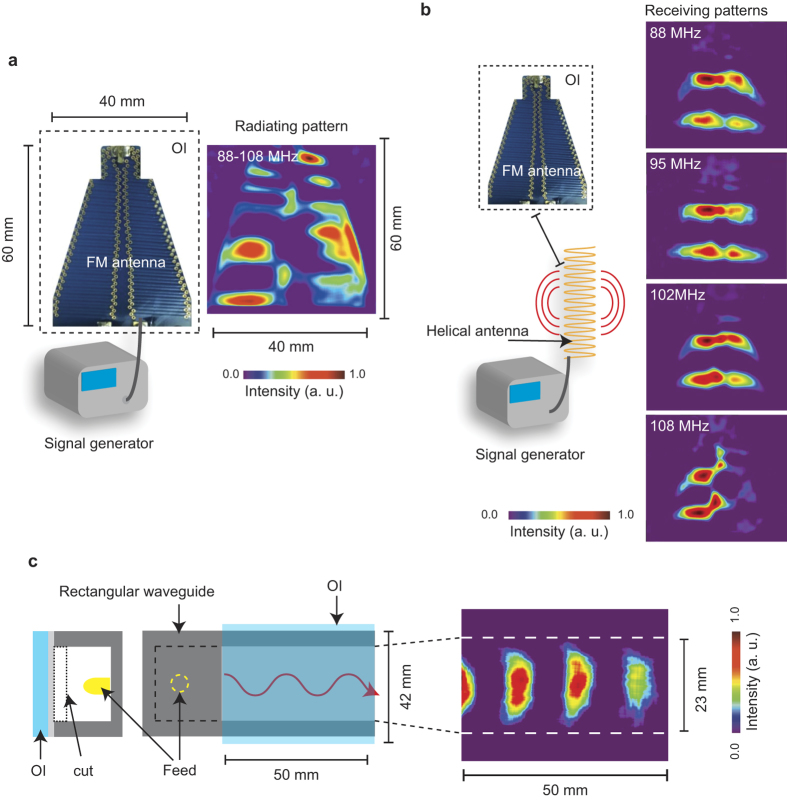
Magnetic microwave near field structures of FM antenna and rectangular wave guide. (**a)** Radiating pattern of FM antenna at 88–108 MHz, where the intensity was normalized to the maximum intensity. (**b**) Receiving pattern of FM antenna at 88, 95, 102, and 108 MHz, where the intensity was normalized to the maximum intensity. The FM signal was generated by a helical antenna connected to a signal generator with an amplifier. (**c**) Propagating pattern of microwave magnetic field in a rectangular waveguide at 10 GHz, where a side of the waveguide was cut and covered by the OI composed of a glass substrate coated by an aluminum thin film (100 nm).

Another advantage of the TEOIM is that the OI is composed of conventional material such as glass and metal thin film. Existing scanning-free OIMs are based on particular single crystal materials having large electro optical and magneto optical effects, such as lithium niobate^15^ and bismuth substituted yttrium iron garnet^11^,^16^. Although such materials can be prepared for a small size, the fabrication of a few tens of centimeter sized OIs will be a difficult, an expensive, or an impractical task. Because the field of view (FOV) of existing OIMs is limited in the size of their OIs, their applications are limited to a few centimeter sized sample or device. However, such a large glass substrate is commercially available, and a coating of a metal thin film on the glass substrate is not a difficult task. Figure 7 shows practical examples demonstrating the excellent FOV of the TEOIM, radiation (a) and receiving (b) magnetic field patterns of a FM-antenna (40 mm by 60 mm) at 88–108 MHz, and propagating microwave magnetic field patterns in a rectangular wave guide (50 mm by 40 mm) at 14 GHz are presented. These examples demonstrate the advantage of TEOIM in the FOV: one can get the whole structure of the MWNF distribution of a large scaled device at once without scanning (or an OI-moving) process. This is an attractive feature to investigate MWNF and its frequency characteristics of a large scale device, where visualized MWNF distribution will be useful to diagnose the device in an operating state.

## Discussion

The results of our research show the functionality of TEOIM for the visualization of the thermal and MWNF distribution imaging. Experimentally, we demonstrated that the TEOIM provides a comparable sensitivity, resolution, and response time to that of existing methods. Because the TEOIM is based on the conventional CCD-based optical microscope system, it can be a practical way that enables one to visualize the spatial structure of the thermal and electromagnetic field under various experimental environments. Although we focused on imaging of thermal and microwave near field in the present study, the experimental results showed how the metal nano-particle and thin film interact differently with the electromagnetic wave at microwave frequency. Therefore, the utility of the TEOIM is not limited to the imaging, but can be extended to characterize the interaction between the material and electromagnetic wave with a wide range of frequency. As the interaction depends on the material’s electromagnetic property, the TEOIM can be applied to visualize the electromagnetic property distribution of a material.

## Methods

### Imaging system

The layout of measurement system is presented in Supplementary Fig. S1a. The system is a typical polarized light microscope composed of two linear sheet polarizers, a CCD (Retiga 2000 rv), and a green light emitting diode (LED; λ = 530 nm). In addition, a liquid crystal modulator (LCM) was introduced to modulate the polarization of the probing light to be a circularly polarized state^30^. All instruments were controlled by a custom computer program, and more detailed descriptions on the system are presented in supplement text S1.

### Heat distribution imaging

#### Measurement

A borosilicate slide glass (Eagle XG; 20 × 20 × 0.5 mm) coated by a platinum thin film (200 nm) was used as the OI for the thermal imaging. The platinum side of the indicator was contacted to the PCB circuit, where the surface of PCB circuit was electrically insulated from the platinum film by an insulating polymer coating. We measured electric resistance of the surface of the conduction line of the PCB circuit by using a multi-meter, and confirmed that the surface was electrically insulated. In addition, the electric resistance of the pt film was larger than 10 Ω (for 2 cm length), while that of the conduction line whose length was much larger than 2 cm was less than 1 Ω. Therefore, we assumed that there was no contribution of an electric current passing through the pt film on the measurement results. A DC electric current was applied to the PCB circuit by Keithley-2400 source meter, and monitored by the present system. Detailed measurement procedures of LB and reconstruction procedures of heat distribution are presented in supplement text S2–4.

### MWNF imaging

#### Fabrication of OIs

For the E-field imaging indicator, an aluminum (Al) thin film (3~5 nm) was deposited on a borosilicate glass substrate (Eagle XG; 20 × 20 × 0.5 mm) by the vacuum thermal evaporation technique. The Al nanoparticles were formed on the glass substrate by annealing the Al thin film at 100 °C for 1 hour. The Al nanoparticles layer was then coated by a PMMA thin film by spin coating, where the PMMA solution was prepared by dissolving it in toluene. After the PMMA coating, the thin film was annealed at 80 °C for 12 hour to polymerize the PMMA layer. For the H-field indicator, a borosilicate glass coated by platinum thin film (200 nm) was used, where the film was prepared by sputtering technique.

#### Measurement

The stepped impedance low pass filter (SILPF), hairpin band pass filter (HBPF), and grounded coplanar waveguide (GCPW) were used as the device under test (DUT). The DUTs were designed for 50 Ω and fabricated on FR4 substrate with patterned copper sheets. Simulations of transmittance and MWNF distribution of the DUTs were conducted by the COMSOL Multiphysics software (see the supplement text S6), and the transmittance measurements were conducted by the network analyzer (E5071b; Agilent technology). For the imaging measurements, the microwave generator (HP-83620A) and the power meter (HP-437B) were used, where the DUTs were connected by 50 Ω BNC-cables to the instruments. The OIs were placed on a device under test (DUT) with an air gap of 0.5 mm to prevent the direct heat conduction of the DUT. The change of LB of the OI was monitored by the present imaging system, and more detailed descriptions on the measurement and analysis procedures are presented in supplement text S1 and Supplement Fig. S2.

## Additional Information

**How to cite this article**: Lee, H. *et al*. Temperature and microwave near field imaging by thermo-elastic optical indicator microscopy. *Sci. Rep.* **6**, 39696; doi: 10.1038/srep39696 (2016).

**Publisher's note:** Springer Nature remains neutral with regard to jurisdictional claims in published maps and institutional affiliations.

## Supplementary Material

Supplementary Material
