# Supplementary material for: Temperature and microwave near field imaging by thermo-elastic optical indicator microscopy
Source: Sci Rep. 2016 Dec 22;6:39696. doi: 10.1038/srep39696 (PMC5177872; doi:10.1038/srep39696)
Supplement: Supplementary Material [file srep39696-s1.pdf]

Temperature and microwave near field imaging by thermo-elastic optical indicator microscopy

Hanju Lee[1], Shant Arakelyan[1,2], Barry Friedman[3], and Kiejin Lee[1,*]

[1]Department of Physics and Basic Science Institute for Cell Damage Control, Sogang University, Seoul 121-742, Republic of Korea

[2]Department of Radiophysics, Yerevan State University, Yerevan 0025, Armenia

[3]Department of Physics, Sam Houston State University, Huntsville, TX 77341, USA

*Corresponding Author: E-mail adress: klee@sogang.ac.kr (K. Lee)

SUPPLEMENTARY INFORMATION

S1. Measurement setup

Linear birefringence (LB) was measured by the polarization modulation technique[1]. Figure S1a shows a schematic diagram of our measurement system, where a green light emitting diode (LED, $\lambda \approx 530$nm) was used as the light source, and two sheet-type polarizers were used as the polarizer and analyzer. The polarization of incident light was modulated by the liquid crystal modulator (LCM) to be in the left-handed or right handed circular polarized state. The LCM acts as a variable linear retarder, whose retardation is modulated by an AC electric voltage generated by a LCM controller so that the polarization state can be modulated by changing a driving voltage without mechanical rotation. The changes of light intensity were monitored by the CCD camera, where two measurements were conducted when the analyzer was aligned to 90 and 45 degrees (Fig. S1b). The analyzer angle was controlled by a stepping motor controller. Operations of all devices and data analysis were conducted by a computer with a custom program.

S2. Jones calculus for thermal stress distribution imaging

From the Fig. S1b, the Jones matrix for each optical component can be expressed as[1,]:

$$J_P = \begin{bmatrix} 1 & 0 \\ 0 & 0 \end{bmatrix}, \quad J_{LCM} = \frac{1}{2}\begin{bmatrix} e^{i\frac{\delta}{2}} + e^{-i\frac{\delta}{2}} & e^{i\frac{\delta}{2}} - e^{-i\frac{\delta}{2}} \\ e^{i\frac{\delta}{2}} - e^{-i\frac{\delta}{2}} & e^{i\frac{\delta}{2}} + e^{-i\frac{\delta}{2}} \end{bmatrix}, \quad J_A = \begin{bmatrix} \cos^2\phi & \cos\phi\sin\phi \\ \cos\phi\sin\phi & \sin^2\phi \end{bmatrix}, \quad \text{(S2.1)}$$

where, δ is the linear birefringence of the LCM, and ϕ is the angle of the analyzer from the x-axis. By assuming no linear and circular dichroism and circular birefringence, the Jones matrix for the sample can be expressed as[2]:

$$J_S = \begin{bmatrix} e^{i\beta}\cos^2\theta + e^{-i\beta}\sin^2\theta & (e^{i\beta} - e^{-i\beta})\cos\theta\sin\theta \\ (e^{i\beta} - e^{-i\beta})\cos\theta\sin\theta & e^{i\beta}\sin^2\theta + e^{-i\beta}\cos^2\theta \end{bmatrix}, \text{(S2.2)}$$

where, β is a linear birefringence induced by thermal stress in the sample, and θ is a angle between the analyzer and the principal stress axis. From the equations (S2.1) and (S2.2), and from the arrangement of optical components shown in Fig. S5c, the intensity of the light reaching the CCD can be calculated as:

$$I = \frac{E_i^2}{4}\left(|A|^2\cos^2\phi + |B|^2\sin^2\phi + \left(A^*B + AB^*\right)\cos\phi\sin\phi\right), \text{(S2.3)}$$

where the E_i is the amplitude of electric field of the incident light, and A and B are:

$$A = j_1\delta_+ + j_2\delta_-, \quad B = j_2\delta_+ + j_1^*\delta_-, \text{(S2.4)}$$

where

$$j_1 = e^{i\beta}\cos^2\theta + e^{-i\beta}\sin^2\theta, \quad j_2 = \left(e^{i\beta} - e^{-i\beta}\right)\cos\theta\sin\theta, \text{(S2.5)}$$

$$\delta_+ = e^{i\frac{\delta}{2}} + e^{-i\frac{\delta}{2}}, \quad \delta_- = e^{i\frac{\delta}{2}} - e^{-i\frac{\delta}{2}}, \text{(S2.6)}$$

By choosing the circular polarized light as a probing beam, and from equations (S3.2.3)-(S3.2.6), the output intensity can be calculated as:

$$I_{\phi=\pi/2,\delta=-\pi/2} = \frac{E_i^2}{2}\left(1 - \sin 2\beta\sin 2\theta\right), \quad I_{\phi=\pi/2,\delta=\pi/2} = \frac{E_i^2}{2}\left(1 + \sin 2\beta\sin 2\theta\right), \text{(S2.7)}$$

$$I_{\phi=\pi/4,\delta=-\pi/2}=\frac{E_i^2}{2}\left(1-\sin 2\beta\cos 2\theta\right),\ \ I_{\phi=\pi/4,\delta=\pi/2}=\frac{E_i^2}{2}\left(1+\sin 2\beta\cos 2\theta\right),\ \text{(S2.8)}$$

and from (S2.7) and (S2.8), and for small *β*,

$$\beta_1=\frac{1}{2}\frac{I_{\phi=\pi/4,\delta=\pi/2}-I_{\phi=\pi/4,\delta=-\pi/2}}{I_{\phi=\pi/4,\delta=-\pi/2}+I_{\phi=\pi/4,\delta=\pi/2}}\cong\beta\cos 2\theta,\ \ \beta_2=\frac{1}{2}\frac{I_{\phi=\pi/2,\delta=\pi/2}-I_{\phi=\pi/2,\delta=-\pi/2}}{I_{\phi=\pi/2,\delta=-\pi/2}+I_{\phi=\pi/2,\delta=\pi/2}}\cong\beta\sin 2\theta,\ \text{(S2.9)}$$

The *β* is related to a difference of stress between the two principle axes, and assuming the thermal stress can be described as the plane stress, the stress tensor can be expressed as :

$$\sigma=\begin{bmatrix}\sigma_x & \sigma_{xy}\\ \sigma_{yx} & \sigma_y\end{bmatrix}=\begin{bmatrix}\sigma_1\cos^2\theta+\sigma_2\sin^2\theta & (\sigma_1-\sigma_2)\cos\theta\sin\theta\\ (\sigma_1-\sigma_2)\cos\theta\sin\theta & \sigma_1\sin^2\theta+\sigma_2\cos^2\theta\end{bmatrix},\ \text{(S2.10)}$$

where, σ_1 and σ_2 are the two principal axis. From equation (S2.9) and (S2.10), and from the stress-optic law, the equation (S2.9) can be related to the stress as:

$$\beta_1=\frac{2\pi dS}{\lambda}\left(\sigma_x-\sigma_y\right),\ \ \beta_2=\frac{2\pi dS}{\lambda}2\sigma_{xy},\ \text{(S2.11)}$$

where *S* is the stress optical constant, *λ* is the wavelength of incident light and *d* is the thickness of the medium.

S3. Constructing the heat source distribution

For plane strain condition in a rectangular Cartesian coordinate system, the thermal stress can be expressed by introducing the stress function[3]:

$$\sigma_x=\frac{\partial^2\Phi}{\partial y^2}+CT,\ \ \sigma_y=\frac{\partial^2\Phi}{\partial x^2}+CT,\ \ \sigma_{xy}=-\frac{\partial^2\Phi}{\partial x\partial y},\ \ C=\frac{\alpha E}{1-2v},\ \text{(S3.1)}$$

where, σ_x, σ_y, and σ_{xy} are the stress components of the stress tensor, *T* is the temperature distribution, *α*, *v*, and *E* are the thermal expansion coefficient, the Poisson's ratio and the elastic modulus of a material, and Φ is the stress function satisfying[3]:

$$\nabla^4\Phi=-\frac{\alpha E}{1-v}\nabla^2 T,\ \text{(S3.2)}$$

The stress function is related to the stress components from (S3.1) as:

$$-\frac{\partial(\sigma_x - \sigma_y)}{\partial x} - 2\frac{\partial \sigma_{xy}}{\partial y} = \frac{\partial(\nabla^2\Phi)}{\partial x},\quad \frac{\partial(\sigma_x - \sigma_y)}{\partial y} - 2\frac{\partial \sigma_{xy}}{\partial x} = \frac{\partial(\nabla^2\Phi)}{\partial y}, \text{ (S3.3)}$$

and it is related to the LB measurement results from (S2.11) and (S3.3) as:

$$\frac{\partial(\nabla^2\Phi)}{\partial x} = -\frac{\lambda}{2\pi dS}\left(\frac{\partial\beta_1}{\partial x} + \frac{\partial\beta_2}{\partial y}\right),\quad \frac{\partial(\nabla^2\Phi)}{\partial y} = \frac{\lambda}{2\pi dS}\left(\frac{\partial\beta_1}{\partial y} - \frac{\partial\beta_2}{\partial x}\right), \text{ (S3.4)}$$

From the stationary heat equation with a heat source, the heat source distribution can be calculated as:

$$q = -\frac{(1-\nu)k}{\alpha E}\left(\frac{\partial^2(\nabla^2\Phi)}{\partial x^2} + \frac{\partial^2(\nabla^2\Phi)}{\partial y^2}\right), \text{ where } q = k\nabla^2 T, \text{ (S3.5)}$$

where, q is the heat source density, and k is the effective thermal conductivity of the platinum coated glass substrate. Finally, from equations (S3.4) and (S3.5), the heat source distribution can be expressed as:

$$q = \frac{\lambda}{2\pi dS}\frac{(1-\nu)}{\alpha Ek}\left(2\frac{\partial^2\beta_2}{\partial x\partial y} + \frac{\partial^2\beta_1}{\partial x^2} - \frac{\partial^2\beta_1}{\partial y^2}\right), \text{ (S3.6)}$$

S4. Averaging and smoothing procedures

Figure S2 shows the measurement process for stationary (a) and time resolved (b) imaging. For each measurement steps, the 100-images were captured by the CCD camera for data and background, and wait steps of ~5 seconds were introduced to saturate and cool down the temperature of the indicator. The time resolved measurement was conducted by capturing continuously with a frame rate of ~13 fps and an exposure time of ~50 ms. The averaging process was conducted by repeating the measurement processes 10~100 times, and therefore, 1,000~10,000 images were averaged. Figure S3c-d show calculated heat source distribution images with varying the averaging and smoothing procedures. Before all differentiations of images, smoothing processes were conducted by taking moving average of the intensity (a).

The smoothed images were then differentiated along the horizontal and vertical direction by calculating the intensity difference between pixels (b). Table S1 shows standard deviation of intensity (N_{rms}) and temperature sensitivity (*TS*) obtained for varying averaging times (AVE), moving average cell size (MAC) and times (MAT).

Table S1: N_{rms} and TS according to interpolation and averaging processes (*).

Current (A)	AVE	MAC	N_{rms} (rad)	*TS* (mK)
0.5	1,000	50	2.5×10^{-6}	55~60
		100	3.2×10^{-6}	11~14
	5,000	50	1.2×10^{-6}	26~29
		100	2×10^{-7}	6~8
	10,000	25	8×10^{-6}	140~145
		50	1.1×10^{-6}	23~25
		100	1.7×10^{-7}	5~7
	15,000	25	5.16×10^{-6}	91~96
		50	7.3×10^{-7}	15~18
		100	1.3×10^{-7}	4~6

*. Ave: Number of images for averaging process; MAC: Radius of moving average cell; N_{rms}: Standard deviation of calculated image; *TS*: Temperature sensitivity (*SITF*=$2.81\sim5.52\times10^{-5}$ rad/K)

S5. Electromagnetic heating mechanisms at microwave frequency

In general, there are three different mechanisms of electromagnetic energy conversion to the heat at microwave frequency: magnetic, dielectric, and resistive losses[4]. For non-magnetic materials, the heating mainly comes from the dielectric and resistive loss. When a loss material is coated on a glass substrate, the temperature change of the glass substrate can be expressed as:

$$\rho C_p \frac{\partial T}{\partial t} - \nabla\left(k\nabla T\right) = q \text{, (S5.1)}$$

where, ρ, k, and C_p are the density, thermal conductivity, and heat capacity of the glass substrate, and q is the heat flux density (or heat source density) of the loss material. For a steady state, one can simplify the equation as:

$$-k\nabla^2 T = q \text{, (S5.2)}$$

where it is assumed that the thermal conductivity of the indicator is homogenous and isotropic.

The generated heat by the microwave depends on the loss property of the material coated on the glass substrate. For a case that when the glass substrate is coated by a dielectric loss material, one can express the generated heat by the dielectric loss per unit volume as:

$$q = \frac{\omega}{2}\varepsilon''|E|^2 \text{, (S5.3)}$$

where and ω is the microwave frequency, ε'' is the imaginary part of the dielectric permittivity of the loss material, E is the electric field strength of the microwave. On the other hand, for a case that a highly conductive metal thin film is coated on the glass substrate ($\sigma \gg \omega\varepsilon$), the resistive losses (or ohmic losses) is responsible mechanism for the heat generation. Then, one can express the absorbed microwave power by the metal thin film as:

$$P_{av} = \int \frac{R_s}{2}|H_t|^2 \, ds \text{, (S5.4)}$$

$$R_s = \sqrt{\frac{\omega\mu}{2\sigma}} = \frac{1}{\sigma\delta_s} \text{, (S5.5)}$$

where, H_t is the microwave magnetic field tangential to the surface of the metal thin film, R_s and δ_s are the surface resistivity and skin depth of the metal thin film, respectively. Then, one can express the heat source density by the resistive losses induced by microwave magnetic field as:

$$q = \frac{P_{av}}{V} = \frac{R_s}{2t}|H_t|^2 \text{, (S5.6)}$$

where t is the thickness of the metal thin film.

S6. Simulations for microwave devices

The transmittances and microwave near field structures of stepped impedance low pass filter (SILPF; Fig. S4a), hairpin band pass filter (HBPF; Fig. S4b), and grounded coplanar waveguide (GCPW; Fig. S4c) were simulated by COMSOL Multiphysics software. In the simulations, a glass plate (20-20-0.5 mm) was included as the indicator, where it was placed on the filters and waveguide with an air gap of 0.5 mm, and simulation parameters for relative permittivity and dielectric loss tangent of the glass plate were 5.5 and 0.0. The boundary conditions of copper patterns were assigned as the 'Perfect E', and relative permittivity and dielectric loss tangent of the DUT-substrate (FR4) were 4.4 and 0.04, respectively.

S7. LB measurement results of the indicators

Figure S5 shows LB measurement results of a bare glass, PMMA(polymethyl methacrylate)/glass, AlNP(aluminum nano-particle)/glass, and PMMA/AlNP/glass samples, where the AlNP and PMMA layers were prepared by thermal evaporation and spin coating techniques, respectively. The samples were placed on the stepped impedance low pass filter (SILPF), and an air gap of 0.5mm was introduced between the samples and SILPF to prevent direct heat conduction between them. The applied microwave frequency and power were 5GHz and 30mW, and 10,000 images were captured and averaged.

S8. Frequency characteristics of HBPF and SILPF devices

Figure S6 shows simulated and measured frequency characteristics of MWNF structure and transmission coefficient (S_{21}) of the (a) HBPF and (b) SILPF. The measured MWNF distribution well describe the behavior of the microwave filters, a strong attenuation of MWNF intensity below the lower cut-off (f_{LC}) and above the higher cutoff (f_{HC}) frequencies, and a strong enhancement within the pass band frequencies, consistent with the

S_{21} measurement results. In particular, the two indicators showed clear selectivity for the electric and magnetic fields at all frequencies.

S9. Spatial resolution

The spatial resolution of an optical microscopy system is determined by the wavelength of the probing light and pixel size of the CCD camera, where the intensity of a pixel corresponds to the information of a physical property at the position. However, because the TEOIM is based on the plane stress analysis, where a stress for a given position is determined by the overall stress distribution in the plane, the information is in the overall intensity relation between pixels rather than an intensity of single pixel.

To verify the resolution limit of the TEOIM, we conducted simulations (COMSOL Multiphysics) for a case that two disk-shaped heat sources (radius=*d*) are separated with a distance of *d* exist in the OI. The first column in Fig. S7 shows simulated optical images as a function of pixel size, and second column shows calculated heat source distribution from the simulated photoelastic images. From the results, one can see that a reasonable resolution that enables to determine the two heat sources distinctly is achieved when the size ratio of the pixel and disks (*d/px*) was larger than 1.25. By considering an ideal case that the dimension of single pixel corresponds to the optical resolution limit, it can be concluded that the minimum detectable dimension of the TEOIM is comparable to that of a typical optical microscope system. The resolution of the TEOIM can be further enhanced by a proper interpolation process. The third column of Fig. S7 shows calculated heat source distribution images with an interpolation process. The interpolation process was conducted as follows: each pixel was divided by a small virtual pixel having a same intensity to real one, and then, the image was smoothed by taking a moving average. As shown in the calculation result with the interpolation process, a reasonable resolution was achieved when the *d/px* is around 0.75.

Therefore, we can conclude that the resolution of the TEOIM is comparable to that of a typical optical microscope system.

S10. References

1. Ishibashi. T. et al., Magneto-optical imaging using polarization modulation method. *J. Appl. Phys.* **100,** 093903 (2006).
2. Xie, X., Simon, J. D. Picosecond circular dichroism spectroscopy: a Jones matrix analysis. *J. Opt. Soc. Am. B* **7,** 1673 (1990).
3. Barron R. F., Barron B. R. *Design for Thermal Stresses.* Ch. 6 (Wiley, 2011).
4. Pozar, D. M. *Microwave Engineering.* 4th ed. New York: Wiley (2011).

Table S2: Comparison table of microwave near field imaging technique

Technique	Imaging type	Imaging sensor	Pixel	Frame rate	Requirements
Parallel electrooptic heterodyne [1]	Parallel sensing	Customized photo diode array	100 by 100 (10,000)	30 frames per second	Electro-optic indicator (ZnTe, $LiNbO_3$). Parallel photonic-heterodyne system. Internal image processing system
Optical scanning (EO) [2]	Optical scanning	Photo diode with spectrum analyzer	40,401-scanning point	3 minutes per frame (4 ms per scanning point)	Electro-optic indicator (ZnTe, $LiNbO_3$) Optical scanning probe system
Optical scanning (MO) [2]	Optical scanning	Photo diode with spectrum analyzer	512-scanning point	4 seconds per frame (7 ms per scanning point)	Magneto-optic indicator (Bi-YIG); Optical scanning probe system Electromagnet
CCD based TEOIM	Parallel sensing	Conventional CCD camera	1024 by 768 (786,432)	15 frame per second	Indicator (pt-coated glass substrate) Polarized light microscope system

[1] Sasagawa K. *et al.* Live Electrooptic Imaging System Based on Ultraparallel Photonic Heterodyne for Microwave Near-Fields. *IEEE Trans. Microw. Theory Tech.* **55,** 2782 (2007).

[2] Masanori T. *et al.* Electromagnetic Field Distribution Measurements using an Optically Scanning Probe System. *Journal of the National Institute of Information and Communications Technology* **53,** 143 (2006).

a

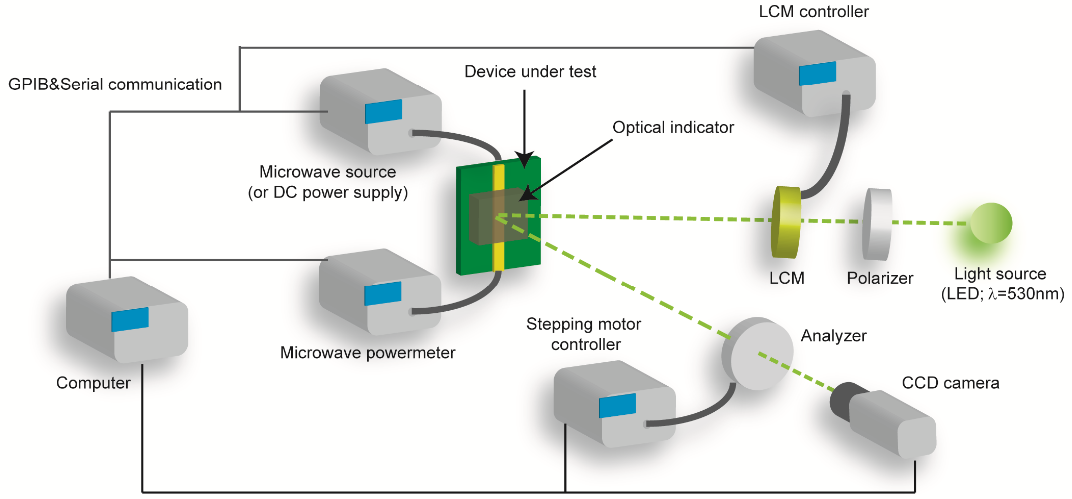

b

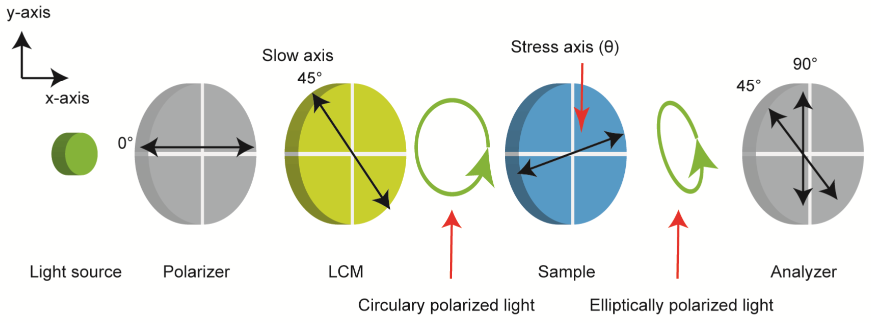

Figure S1: a. Illustration of measurement setup. **b.** Arrangement of optical components.

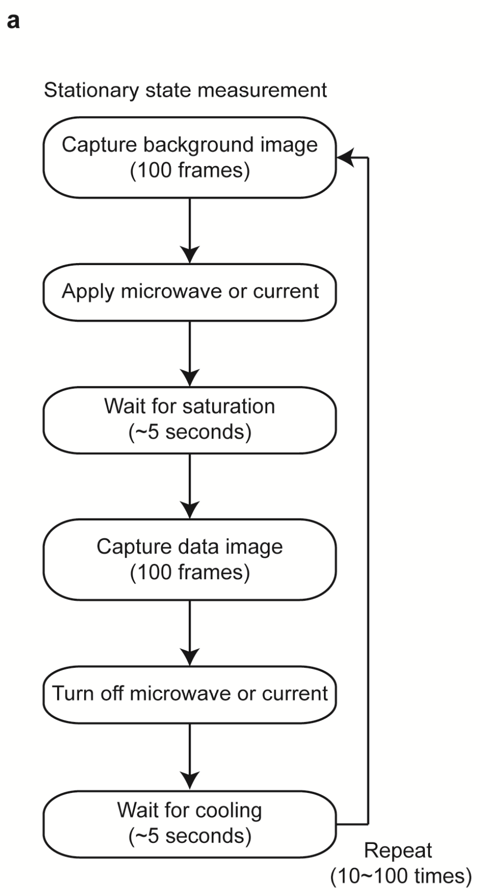

Figure S2: Illustration of measurement processes: **a.** stationary state measurement; **b.** time resolved measurement.

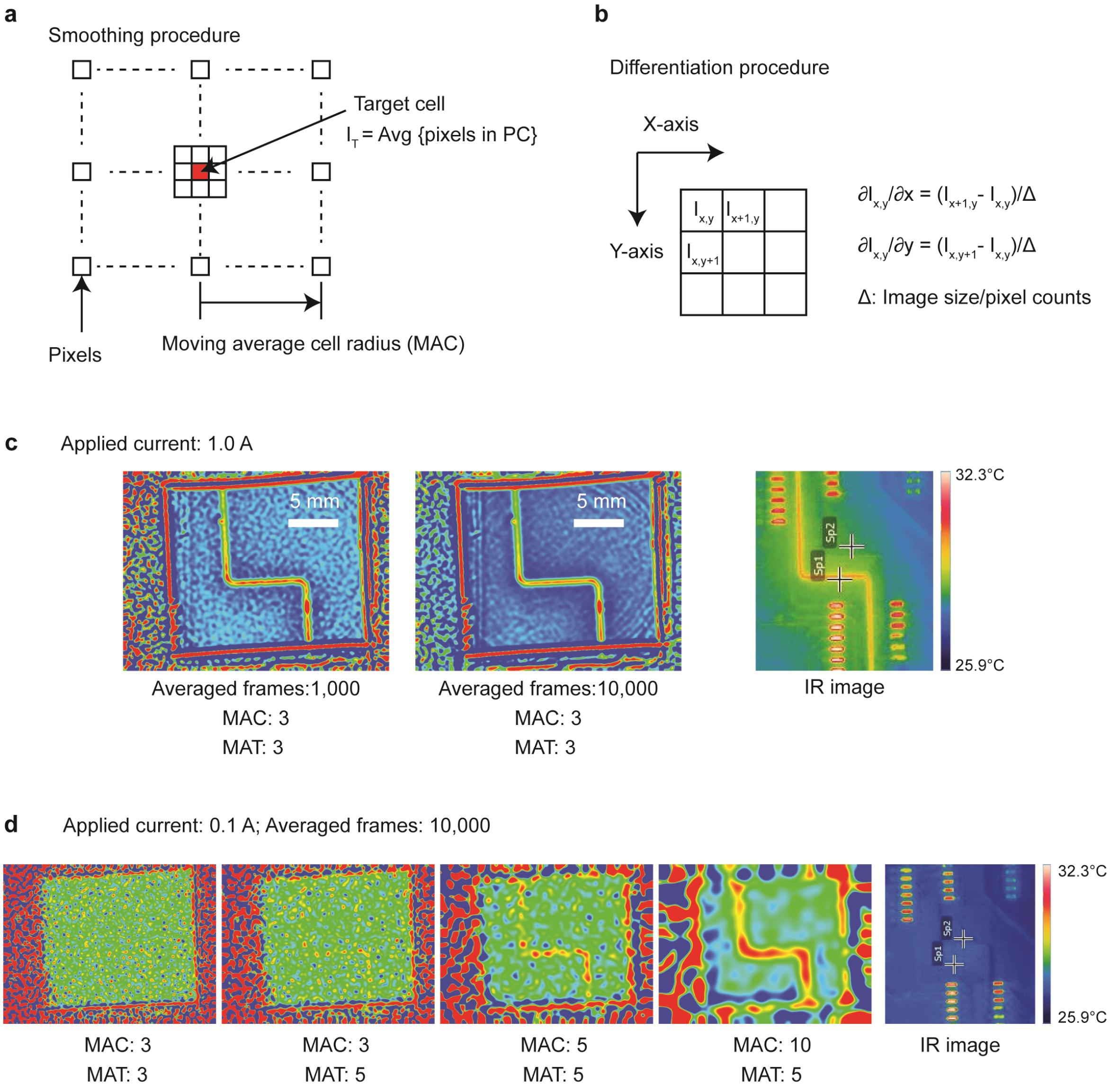

Figure S3: a. Illustration for the smoothing procedure. **b.** Illustration for the differentiating procedure. **c.** Calculated heat source distribution images with 1,000 (first image) and 10,000 averaging (second image). The IR image of the PCB circuit is also presented (last image). **d.** Calculated heat source distribution images with various moving averaging cell size and times.

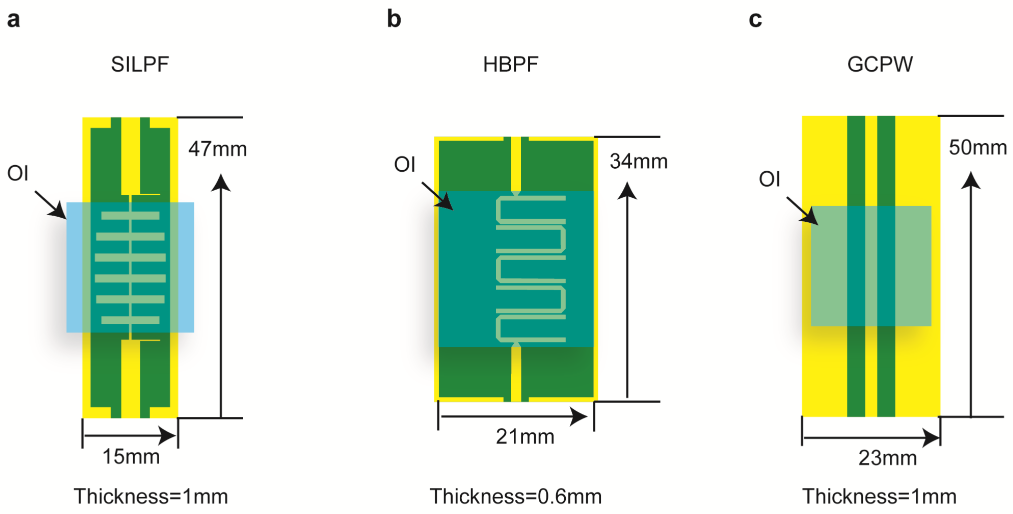

Figure S4: Illustrations of the microwave devices used in the microwave near-field imaging experiments. **a.** Stepped impedance low pass filter (SILPF). **b.** Hair-pin band pass filter (HBPF). **c.** Grounded co-planar waveguide (GCPW).

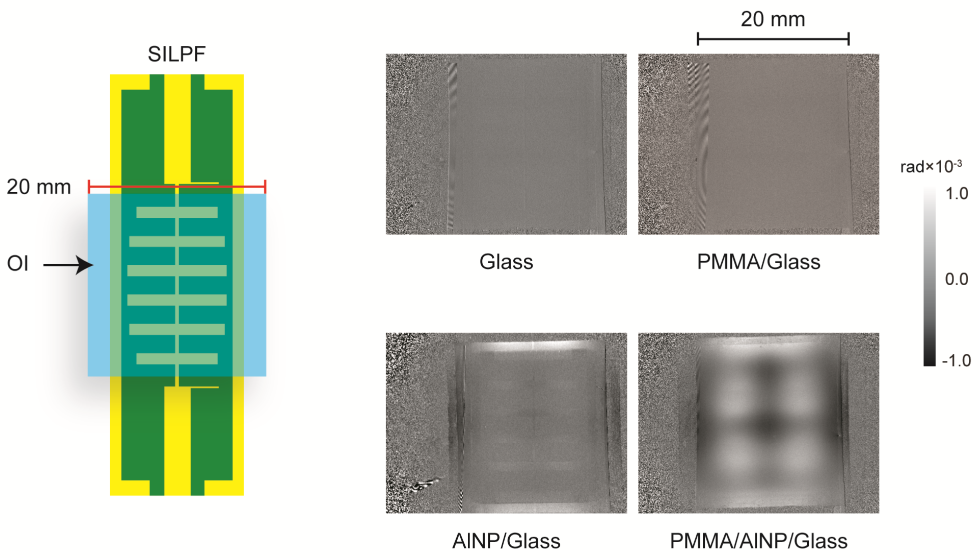

Figure S5: Photoelastic images for bare glass, PMMA coated glass, AlNP coated glass, and PMMA/AlNP coated glass substrates.

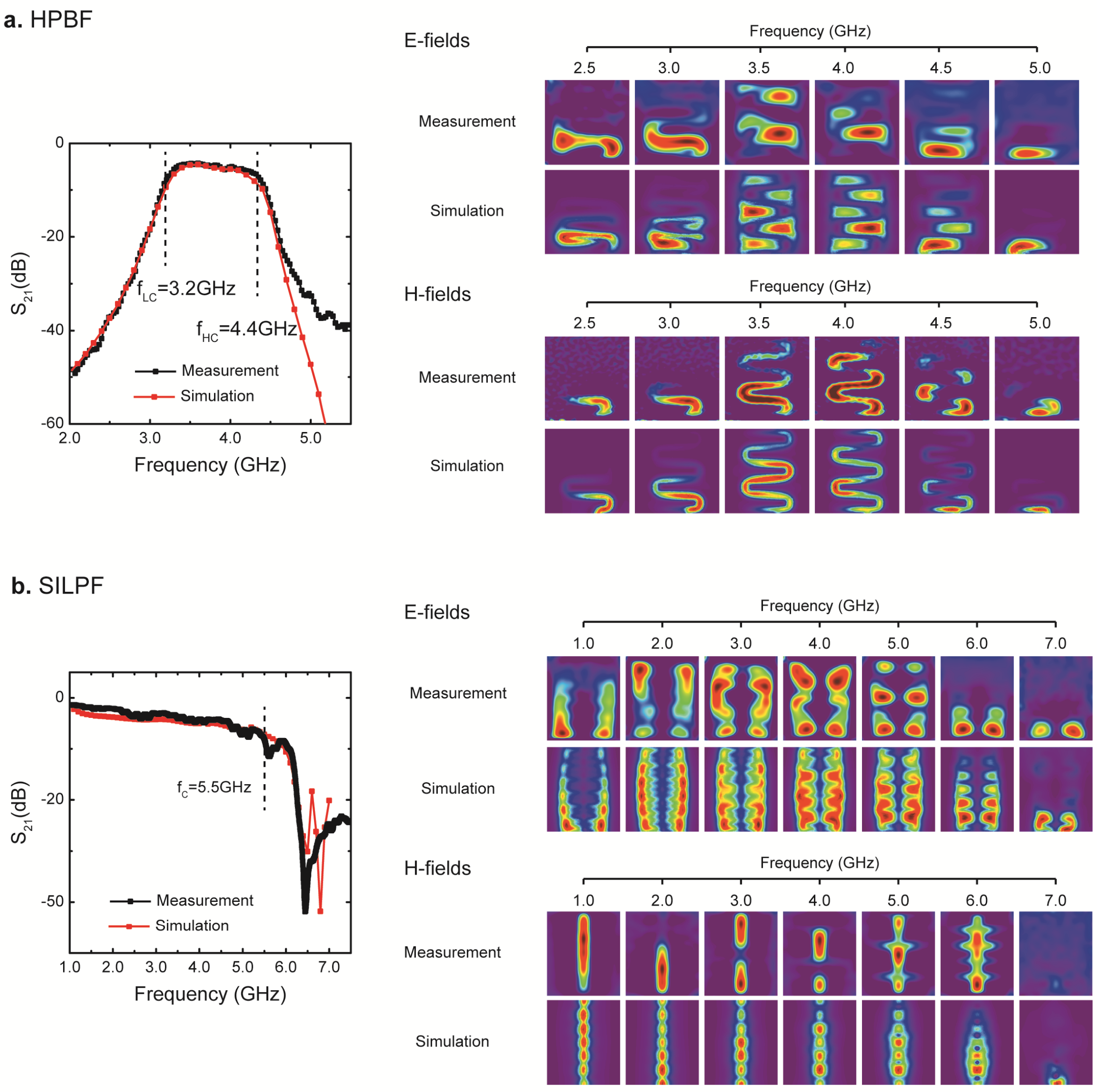

Figure S6: Frequency characteristics of the electric and the magnetic near field structures of HBPF and SILPF devices visualized by the TEOIM with simulation results. Measured (black) and simulated (red) microwave transmittance (S_{21}) with MWNF structures as a function of applied microwave frequency for HBPF (a) and (b) SILPF are also presented. The S_{21} measurements were conducted by a network analyzer. The dashed lines indicate the lower (f_{LC}) and higher (f_{HC}) cut-off frequencies of HBPF, and the cut-off frequency (f_c) of the SILPF.

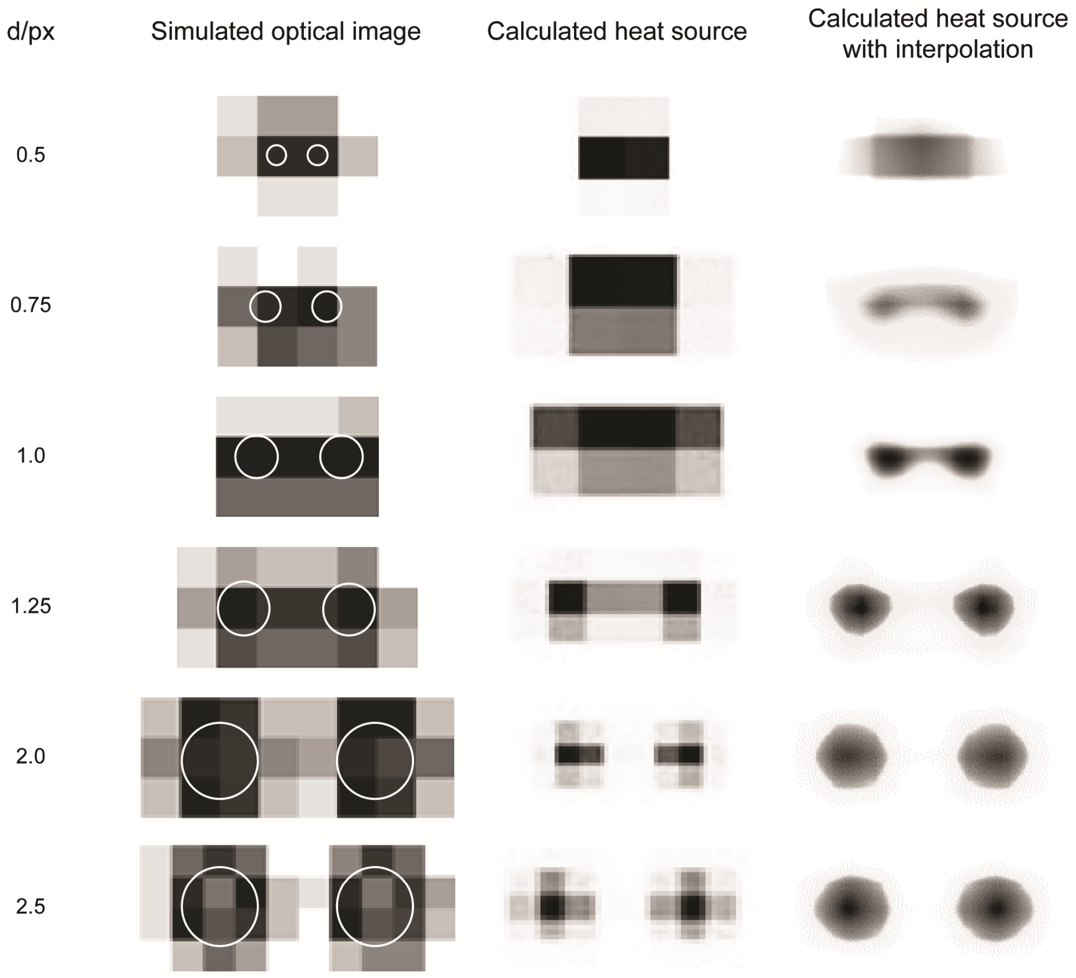

Figure S7: Simulated images of optical (left) and calculated heat source distribution without (middle) and with (right) interpolation process. White circles appeared in simulated optical images (left) indicate the position of heat sources.
